# Efficient yeast surface-display of novel complex synthetic cellulosomes

**DOI:** 10.1186/s12934-018-0971-2

**Published:** 2018-08-07

**Authors:** Hongting Tang, Jiajing Wang, Shenghuan Wang, Yu Shen, Dina Petranovic, Jin Hou, Xiaoming Bao

**Affiliations:** 1 State Key Laboratory of Microbial Technology, Shandong University, Binhai Road 72, Jimo, Qingdao, 266237 People’s Republic of China; 2 Shandong Provincial Key Laboratory of Microbial Engineering, Qi Lu University of Technology, Jinan, 250353 People’s Republic of China; 3 Department of Biology and Biological Engineering, Chalmers University of Technology, Kemivagen 10, 41296 Gothenburg, Sweden

## Abstract

**Background:**

The self-assembly of cellulosomes on the surface of yeast is a promising strategy for consolidated bioprocessing to convert cellulose into ethanol in one step.

**Results:**

In this study, we developed a novel synthetic cellulosome that anchors to the endogenous yeast cell wall protein a-agglutinin through disulfide bonds. A synthetic scaffoldin ScafAGA3 was constructed using the repeated N-terminus of Aga1p and displayed on the yeast cell surface. Secreted cellulases were then fused with Aga2p to assemble the cellulosome. The display efficiency of the synthetic scaffoldin and the assembly efficiency of each enzyme were much higher than those of the most frequently constructed cellulosome using scaffoldin ScafCipA3 from *Clostridium thermocellum*. A complex cellulosome with two scaffoldins was also constructed using interactions between the displayed anchoring scaffoldin ScafAGA3 and scaffoldin I ScafCipA3 through disulfide bonds, and the assembly of secreted cellulases to ScafCipA3. The newly designed cellulosomes enabled yeast to directly ferment cellulose into ethanol.

**Conclusions:**

This is the first report on the development of complex multiple-component assembly system through disulfide bonds. This strategy could facilitate the construction of yeast cell factories to express synergistic enzymes for use in biotechnology.

**Electronic supplementary material:**

The online version of this article (10.1186/s12934-018-0971-2) contains supplementary material, which is available to authorized users.

## Background

Lignocellulosic bioethanol has been widely proposed as a generate sustainable and environmentally-friendly biofuel to replace or complement bioethanol derived from starch or sugar [1, 2]. Consolidated bioprocessing (CBP), which combines cellulase production, cellulose hydrolysis and fermentation into a single process, is currently considered a promising method for bioethanol production owe to its potential low cost and high efficiency [3, 4]. *Saccharomyces cerevisiae*, a traditional ethanol producer, is an attractive candidate for the construction of CBP cell factories [5, 6]. However, as *S. cerevisiae* does not naturally produce cellulases, it cannot degrade cellulose into fermentable sugars.

Heterologous cellulases have been expressed in *S. cerevisiae* using three approaches: secreting the enzymes to the extracellular space, displaying the enzymes on the cell surface and assembling multienzymatic complexes on the cell surface in a structure named the cellulosome [7–10]. Cellulosomes from anaerobic bacteria contain noncatalytic scaffoldins consisting of one or more cellulose-binding domains (CBD) and repeated cohesin domains that can dock different cellulases, tagged with a dockerin domain, in the presence of Ca^2+^ ions [11]. The highly ordered structure facilitates enzyme–enzyme synergy and host–enzyme–substrate proximity which allows efficient cellulose hydrolysis [11, 12]. Previously, heterologous cohesin–dockerin pairs from anaerobic bacteria, such as *Clostridium thermocellum*, *C. cellulolyticum* and *Ruminococcus flavefaciens*, were used for the self-assembly of cellulosome on the surface of yeast [13, 14]. Efforts have also been made to improve the functional efficiency of yeast cellulosomes. For example, the display level of scaffoldin was increased by replacing the the display system with that from the galactose induced a-agglutinin to constitutively expressed α-agglutinin [15]. However, the number of cells with efficient display was still relatively low. Other approaches such as increasing the cohesin by using two scaffoldins instead of one could improve the assembly efficiency of cellulases [12, 16]. Despite progress in this field, the direct conversion of cellulose into ethanol using yeast cellulosomes remains challenging, while the limitations including the low scaffoldin display level, the inefficient self-assembly of cellulases on scaffoldin, and the low activity of cellulases.

In the cellulosome, the dockerin domains of the cellulase and cohesin domains of the scaffoldin interact through non-covalent bonds, including significant hydrophobic interactions and complementary hydrogen bonding [17]. While most studies have directly introduced these dockerins and cohesins into *S. cerevisiae*, a previous study reported using the Z domain protein A and the Fc domain of human IgG as cohesion and dockerin, respectively, to regulate the assembly ratio of cellulosomal cellulases [19]. The Z domain of staphylococcal protein A in *Staphylococcus aureus* is responsible for binding the Fc-portion of immunoglobulin G (IgG) through non-covalent bonds, which plays an important role in qualitative and quantitative immunology [18]. This study indicated that protein pairs which can interact with each other, other than traditional cellulosomal cohesin–dockerin, could also be used for cellulosome assembly.

The *S. cerevisiae* cell adhesion protein a-agglutinin displayed on the cell surface is composed of surface cell wall anchorage subunit Aga1p and cell–cell binding subunit Aga2p, which is linked to Aga1p by the formation of two disulfide bonds [20, 21]. a-Agglutinin has been widely used as the yeast surface display system for anchoring heterologous proteins in numerous applications, including vaccine and antibody development, library screening, bioconversion, and biosorption [22–24]. The precursor of Aga1p contains a secretory signal peptide, a domain rich in threonine and serine residues and the glycosyl phosphatidylinositol (GPI) anchor [25]. The N-terminal 149-residue fragment of Aga1p, named tAga1p, is responsible for the formation of a disulfide-linked complex with Aga2p [26]. Thus, tAga1p and Aga2p could be a potential protein pair for the assembly of protein complexes, similar to the role of cohesin and dockerin.

In this study, we constructed novel synthetic cellulosomes using the Aga1p and Aga2p protein pair display system. The display level of novel scaffoldin ScafAGA containing repeated tAga1p domains was significantly improved compared with the benchmark display level of scaffoldin ScafCipA3 from *C. thermocellum*. The assembly of cellulases through disulfide bonds was also more efficient than that thought non-covalent bonds. A complex synthetic cellulosome with two scaffoldins, using ScafAGA as anchor scaffoldin II and ScafCipA3 as scaffoldin I, was also successfully constructed. This interaction system of Aga2p and repeated tAga1p domains can provide multiple docking units for heterologous proteins to improve their display efficiency, and therefore facilitate yeast to express synergistic enzyme systems in the future.

## Results

### Design of synthetic scaffoldins and cellulosomes

**Fig. 1:**
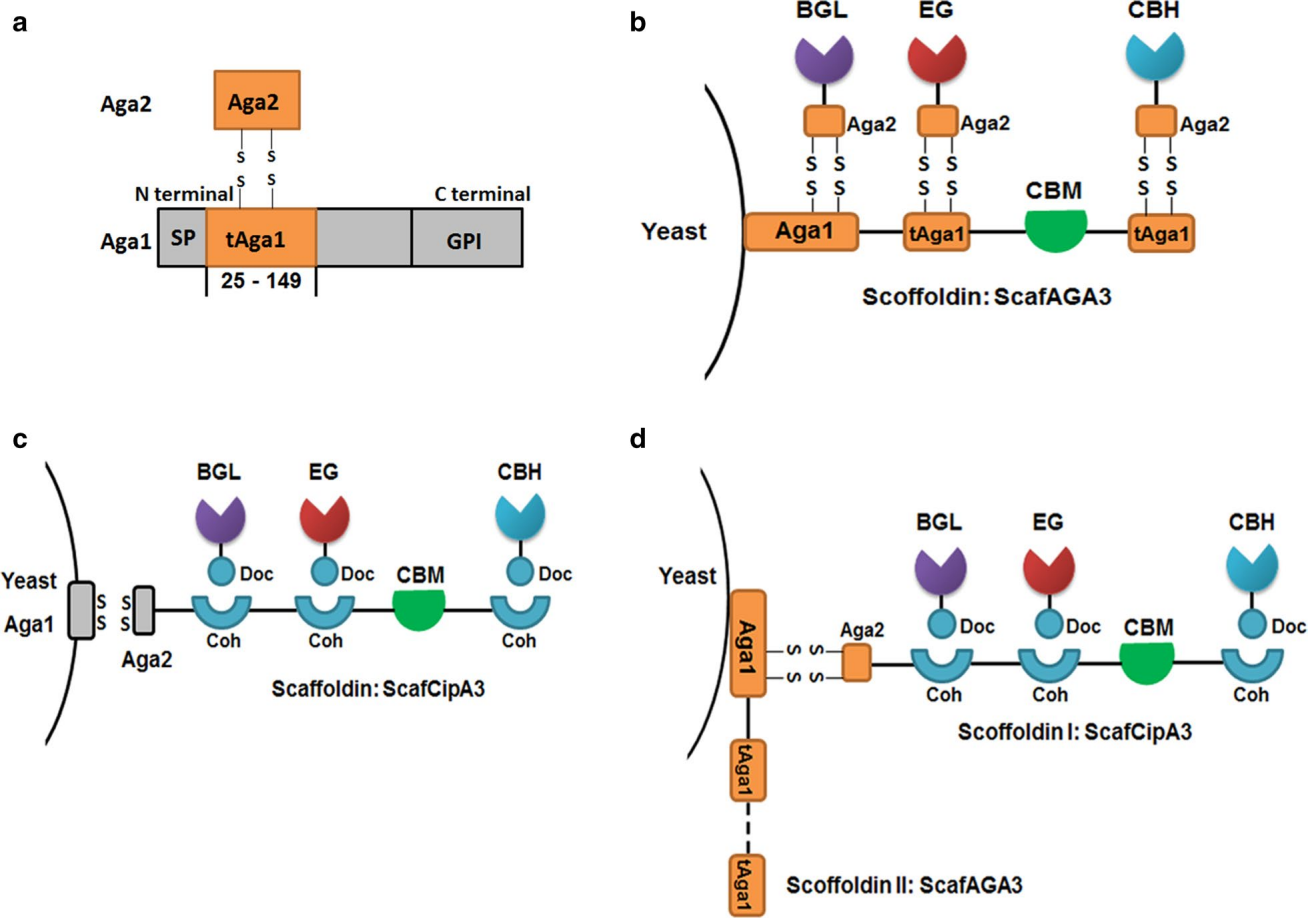
Design of self-assembling cellulosome on the yeast surface. **a** Interaction between Aga1p and Aga2p via disulfide bonds. **b** Assembly of cellulases on the surface-displayed synthetic scaffoldins through covalent disulfide bonds. AGA2s, used as dockerins; tAGA1s, used as cohesins; GPI, the glycosyl phosphatidylinositol anchor; SP, signal peptide; BGL, β-glucosidase; EG, endoglucanase; CBH, exoglucanase; CBM, carbohydrate-binding module. **c** Assembly of cellulosome using scaffoldins from *C. thermocellum*. **d** The assembly of complex cellulosomes with two scaffoldins through covalent disulfide bonds

Herein, instead of cohesins and dockerins (Coh–Doc) from bacterial cellulosomes, the protein pair of Aga1p and Aag2p was used for synthetic yeast cellulosome construction, which was assembled through covalent (disulfide) bonds (Fig. 1). The repeated N-terminal fragment of Aga1p (amino acids 25–149, named tAga1p) was fused with a CBD domain from *Trichoderma reesei* and displayed on the yeast cell surface through the Aga1p C-terminal domain (amino acids 150–701) to construct a synthetic scaffoldin named ScafAGA3 (Fig. 1a). This synthetic scaffoldin was used as the primary scaffoldin or anchor scaffoldin. Aga2 was either fused with secreted cellulases directly to construct simple cellulosomes (Fig. 1b) or fused with scaffoldin from *C. thermocellum* ScafCipA3, which previously described the benchmark cellulosome (Fig. 1c), and then assembled into cellulases through cohesin–dockerin interactions to construct complex cellulosomes (Fig. 1d).

**Fig. 2:**
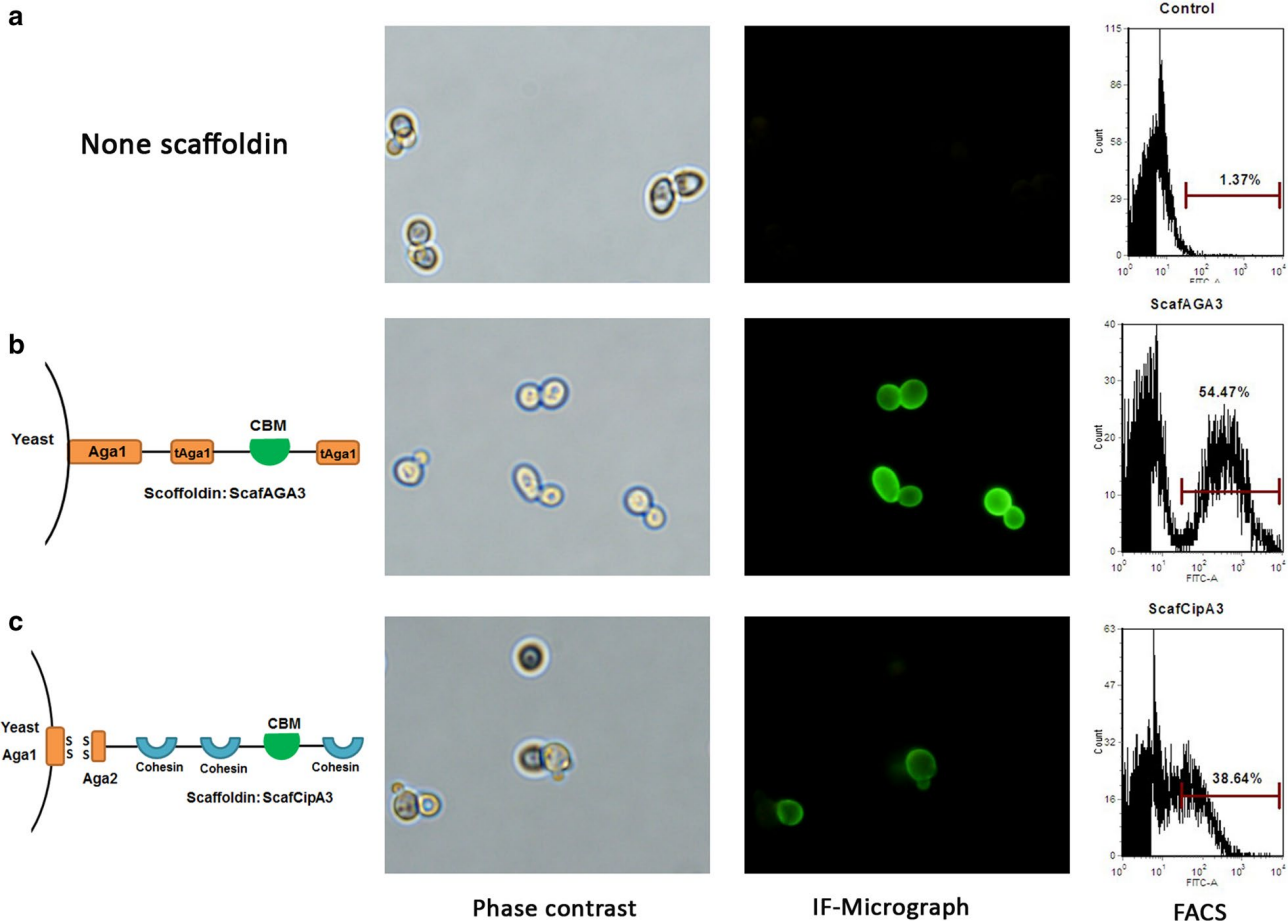
Immunofluorescence micrographs and FACS analysis of scaffoldin surface display. **a** The control strain with empty plasmid expression. **b** Strains displaying the synthetic scaffoldin ScafAGA3. **c** Strains displaying the traditional scaffoldin ScafCipA3. Control represented the strain without staining. Results are representative of three independent experiments. Anti-V5 antibody was used to detect the scaffoldin display efficiency. The *x*-axis (FITC-A) represents the expression levels of scaffoldin as measured using the fluorescence intensity of green fluorescein

We first detected whether tAga1p was able to interact with Aga2. The tAga1p domain was fused with another anchor protein, Sed1p [27], and functionally docked a *C. thermocellum* endoglucase fused to Aga2 (*Ct*-aCelA) on the cell wall surface, demonstrating successful assembly through tAga1p and Aga2 (Additional file 1: Fig. S1). The display efficiency of synthetic scaffoldin ScafAGA3 (three repeat tAga1 units and a C-terminal Aga1p fragment) was then determined. The display level of scaffoldins was determined by immunofluorescence labeling using the anti-V5-FITC antibody, which recognized the V5 tag behind scaffoldin ScafAGA3, through microscopy and flow cytometry analyses (FACS). The ScafAGA3-expressing cells generated strong green fluorescence (Fig. 2b). In contrast, the cells with empty plasmid were not immunostained (Fig. 2a). The FACS results showed that 54.6% of cells were positively stained in the ScafAGA3 expressing strain (Fig. 2b). As shown in Fig. 2c, ScafCipA3 was also successfully displayed on the cell wall surface, with 38.6% of the cells positively stained. Therefore, the proportion of immunostained cells in the ScafAGA3-expressing strain was 41.3% higher than that of the ScafCipA3-expressing strain. These results demonstrated that synthetic scaffoldin ScafAGA3 had a better display level than traditional scaffoldin ScafCipA3.

### Selection of cellulosomal cellulases

**Fig. 3:**
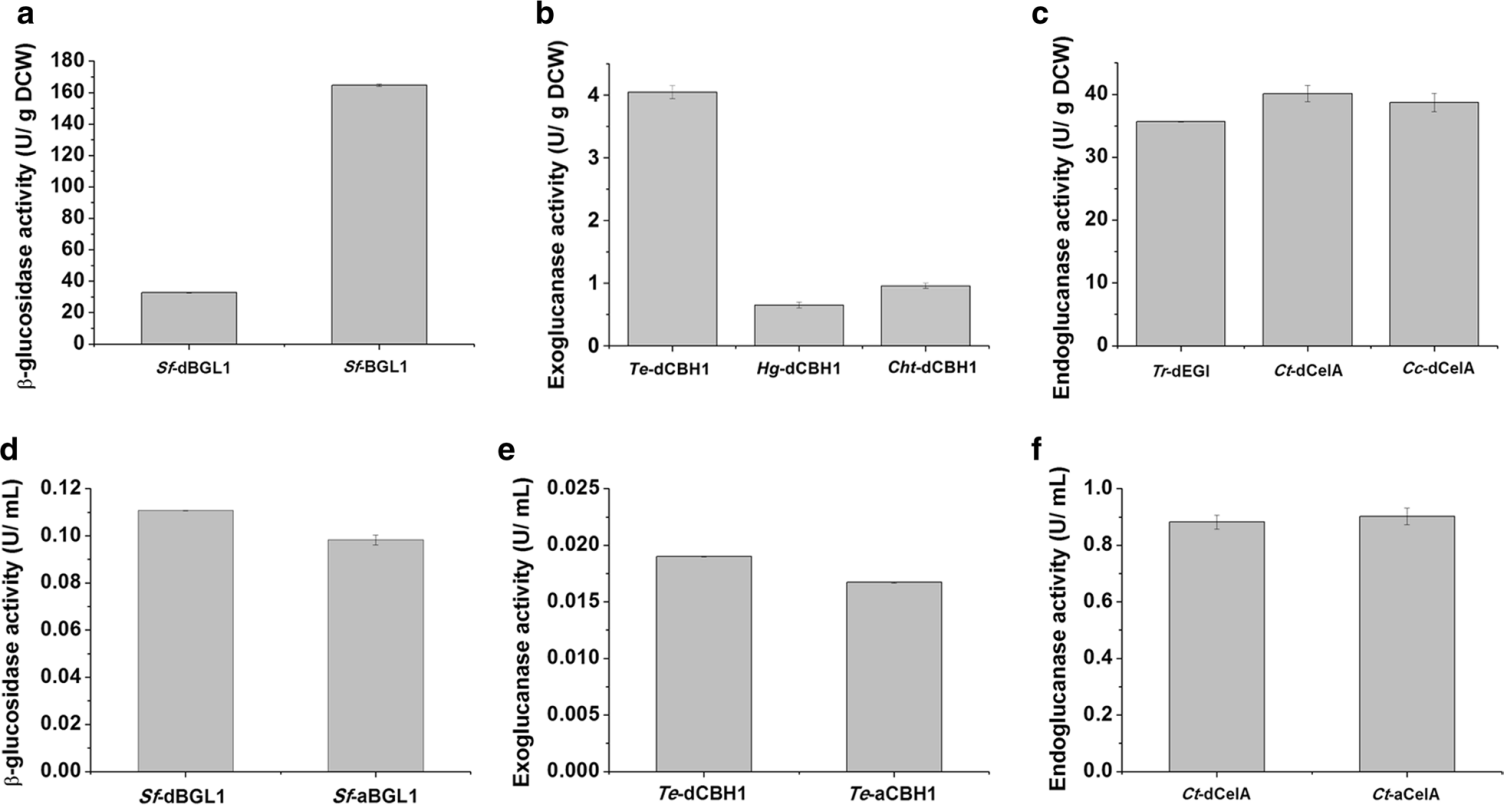
Extracellular activity of cellulosomal cellulases. **a** Activity of β-glucosidase with and without dockerin. **b** Comparison of different exoglucanase activities with dockerin. *Te*, *T. emersonii*; *Hg*, *Humicola grisea*; *Ct*, *Chaetomium thermophilum*. **c** The comparison of different endoglucanase activity with dockerin. *Tr*, *T. reesei*; *Ct*, *C. thermocellum*; *Cc*, *Clostridium cellulolyticum*. Activity of **d** β-glucosidase, **e** exoglucanase and **f** endoglucanase fused with dockerin (d) or AGA2 (a). Data are the mean values of two repeats ± standard deviation

We expressed cellulases, including β-glucosidase, exoglucanase and endoglucanase from different origins in *S. cerevisiae* and compared their activities. In our previous study, *Saccharomycopsis fibuligera* β-glucosidase (*Sf*-BGL1) showed higher activity than β-glucosidases from *Aspergillus niger* in *S. cerevisiae* [10]. Therefore, *S. fibuligera* β-glucosidase was fused with the dockerin for cellulosome assembly. The extracellular activity of recombinant BGL1 (*Sf*-dBGL1) was successfully detected, but fusion with *C. thermocellum* XynC-dockerin significantly decreased the β-glucosidase activity (Fig. 3a). Furthermore, the extracellular activities of the three exoglucanases from *Talaromyces emersonii* (*Te*-dCBH1), *Humicola grisea* (*Hg*-dCBH1) and *Chaetomium thermophilum* (*Cht*-dCBH1) fused with CelS-dockerin derived from *C. thermocellum* exoglucanases were compared and *Te*-dCBH1 showed the highest extracellular activity (Fig. 3b). The activities of endoglucanases from *Trichoderma reesei* (*Tr*-dEG1), *C. thermocellum* (*Ct*-dCelA) and *C. cellulolyticum* (*Cc*-dCelA) fused with the CelA-dockerin derived from *C. thermocellum* endoglucanases were also compared, with *Ct*-dCelA having the highest activity (Fig. 3c). Therefore, we chose *Sf*-dBGL1, *Te*-dCBH1 and *Ct*-dCelA for cellulosome assembly. In addition to fusing the cellulases with dockerin from *C. thermocellum*, these three cellulases were also fused with Aga2p for synthetic cellulosome assembly. The extracellular activities of the Aga2p-fused cellulases, *Sf*-aBGL1, *Te*-aCBH1 and *Ct*-aCelA, were similar to those of the respective dockerin-fused enzymes (Fig. 3d–f).

### Functional assembly of cellulases on synthetic scaffoldins through disulfide bonds

**Fig. 4:**
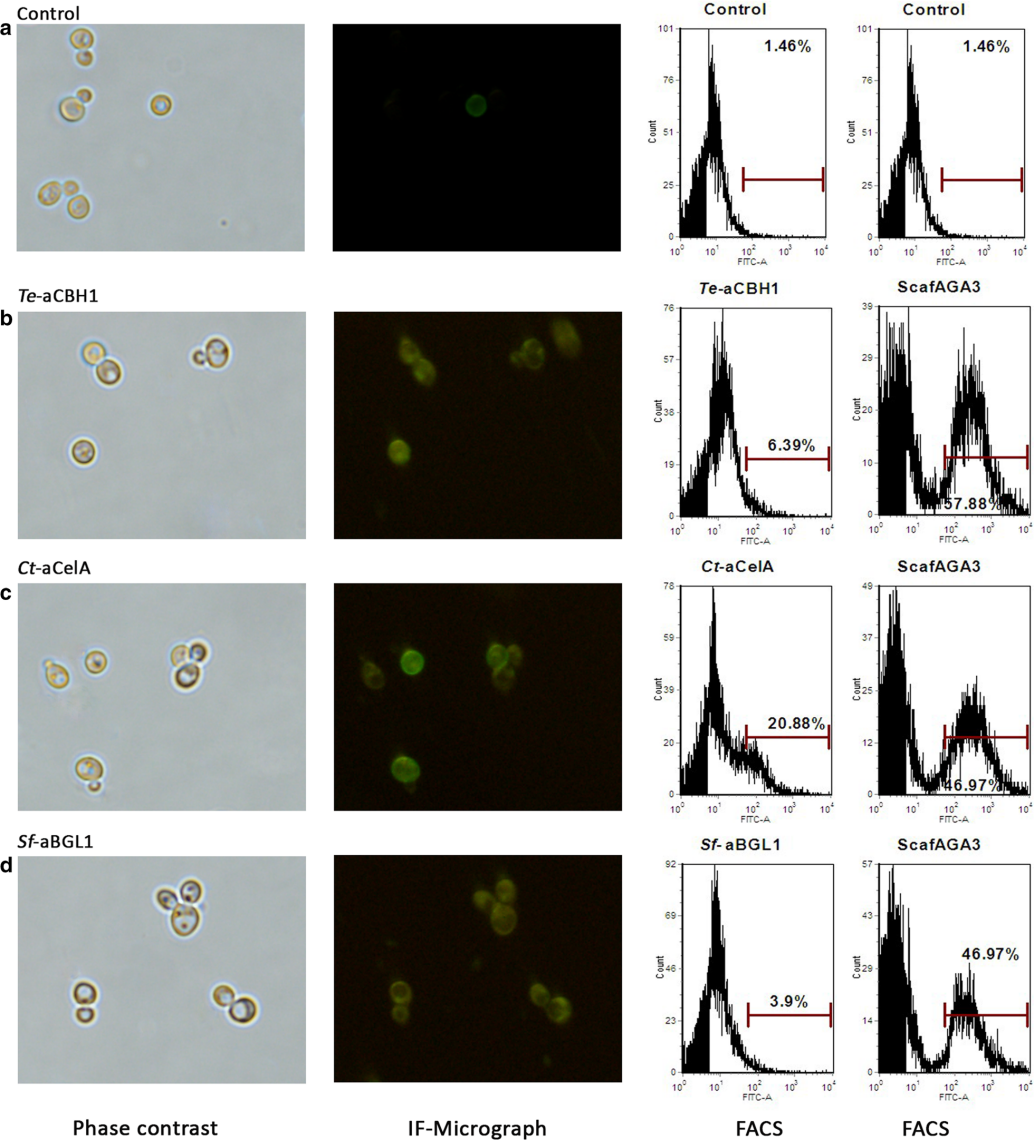
Immunofluorescence micrographs and FACS analysis of each enzyme self-assembly through covalent disulfide bonds. **a** Control strain with expression of empty plasmids. **b** Assembly of *Te*-aCBH1 onto ScafAGA3 on yeast cell wall. **c** Assembly of *Ct*-aCelA onto ScafAGA3 on yeast cell wall. **d** Assembly of *Sf*-aBGL1 onto ScafAGA3 on yeast cell wall. Anti-DDDDK antibody was used to detect the assembly of BGL1 and CBHI, and anti-Myc tag antibody was used to detect the assembly of CelA. Control represents the strain without staining. Results are representative of two independent repeat experiments

To examine whether cellulases could assemble on synthetic scaffoldin ScafAGA3 through disulfide bonds, *Te*-aCBH1, *Ct*-aCelA and *Sf*-aBGL1 were separately co-expressed with ScafAGA3. The fluorescence of the assembled cellulases was clearly observed in strains where ScafAGA3 was co-expressed with *Te*-aCBH1, *Ct*-aCelA and *Sf*-aBGL1 (Fig. 4). The results of FACS analysis further confirmed the self-assembly of the three cellulases with ScafAGA3, and the stained populations of ScafAGA3 with *Te*-aCBH1, *Ct*-aCelA and *Sf*-aBGL1 co-expressing strains were 6.4%, 20.9% and 3.9%, respectively. Co-expression of *Te*-aCBH1 or *Ct*-aCelA did not decrease the display level of ScafAGA3, while *Sf*-aBGL1 slightly decreased the display level. These results confirmed that the three cellulases were successfully assembled on ScafAGA3. In contrast, no positively stained population was detected in the strains co-expressing *Te*-dCBH1, *Ct*-dCelA and *Sf*-dBGL1 with ScafCipA3. Positively stained cells were detected only when the fermentation supernatant containing *Te*-dCBH1, *Ct*-dCelA and *Sf*-dBGL1 was concentrated and manually added the cells expressing ScafCipA3 for cellulosome assembly (Additional file 2: Fig. S2). These findings demonstrated that the assembly efficiency of cellulases through disulfide bonds to ScafAGA3 was much higher than the non-covalent assembly to ScafCipA3 using the cohesin–dockerin protein pair (*Te*-aCBH1 vs. *Te*-dCBH1: 6.39% vs. 1.80%; *Ct*-aCelA vs. *Ct*-dCelA: 20.88% vs. 1.58%; *Sf*-aBGL1 vs. *Sf*-dBGL1: 3.90% vs. 1.58%). Furthermore, the yeast cells displaying ScafAGA3 assembled cellulosome produced 0.89 g/L ethanol from phosphoric acid swollen cellulose (PASC) at 72 h, which were threefold higher than the ethanol production from cells displaying the ScafCipA3 assembled cellulosome (Additional file 3: Fig. S3). These results clearly showed that the higher displaying efficiency and assembly efficiency of the synthetic cellulosome improved enzyme loading and ethanol production.

### Functional assembly of complex cellulosome through disulfide bonds

**Fig. 5:**
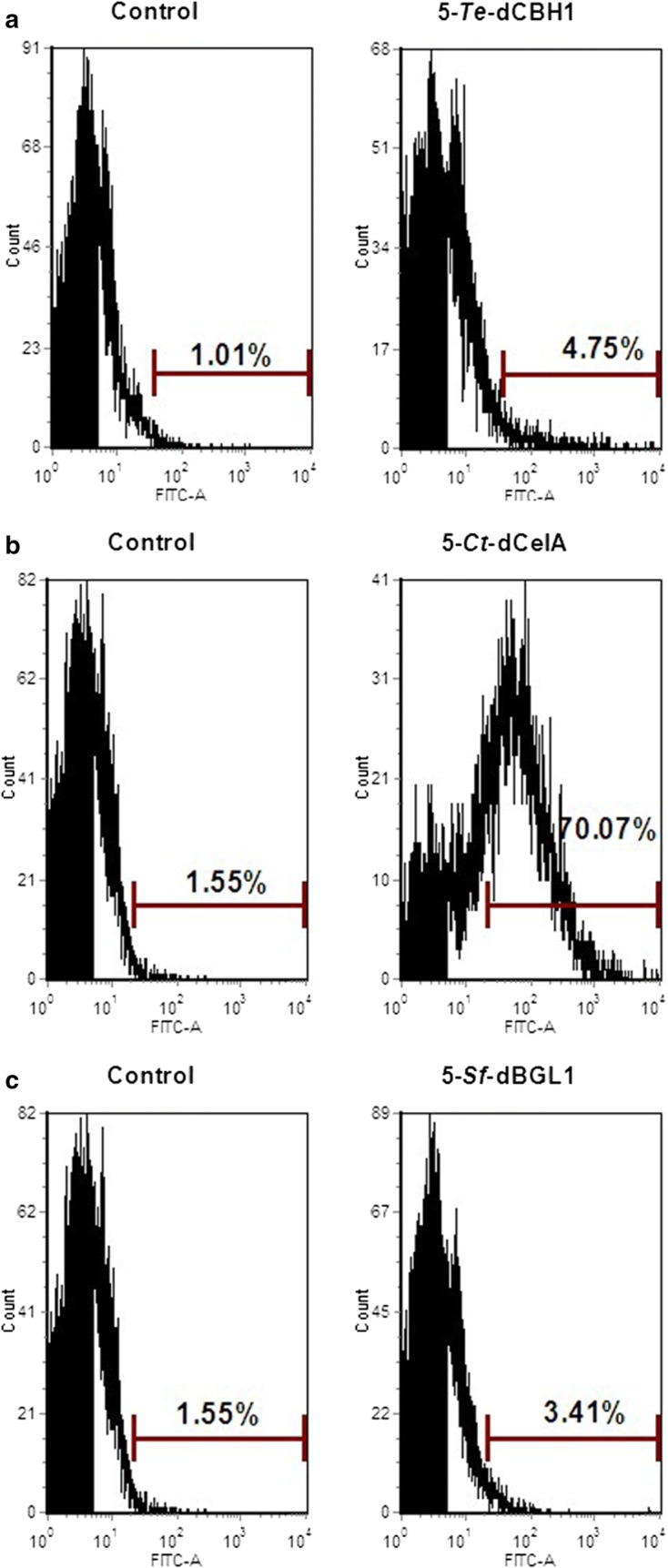
FACS analysis of self-assembly of **a** *Te*-dCBH1, **b** *Ct*-dCelA and **c** *Sf*-dBGL1 using two scaffoldins. Cellulases were assembled on ScafCipA3, and ScafCipA3 was assembled on ScafAGA3 on the yeast cell surface. Control represents the strain without staining. The fermentation supernatant containing *Te*-dCBH1, *Ct*-dCelA and *Sf*-dBGL1 was concentrated about tenfold, respectively, and OD_600_ of 5 of scaffoldin expressing strain was added to the concentrated enzymes for complex cellulosome assembly. Anti-DDDDK antibody were used to detect the assembly of BGL1 and CBHI, and the anti-Myc tag antibody was used to detect the assembly of CelA. Results are representative of two independent repeat experiments

In addition to cellulase assembly with ScafAGA3 through disulfide bonds, we also constructed complex cellulosomes with two scaffoldins through the interaction of tAga1p and Aga2p. Synthetic scaffoldin ScafAGA3, which was displayed on the cell surface, was used as anchoring scaffoldin, and the ScafCipA3 fused with Aga2p was used as the scaffoldin I for cellulase assembly. ScafCipA3 interacted with ScafAGA3 through Aga1p–Aga2p protein pair interaction (Fig. 1d). *Te*-dCBH1, *Ct*-dCelA and *Sf*-dBGL1 were secreted extracellularly to dock with ScafCipA3 for complex cellulosome construction. The cellulases successfully assembled on ScafCipA3 when the enzymes were concentrated, which demonstrated that the complex cellulosome with two scaffoldins can self-assemble on the cell surface (Fig. 5). However, we hypothesized that low expression of the cellulases could affect cellulosome self-assembly on yeast.

### Optimization of cellulase activity and scaffoldin length increased ethanol production from PASC

**Fig. 6:**
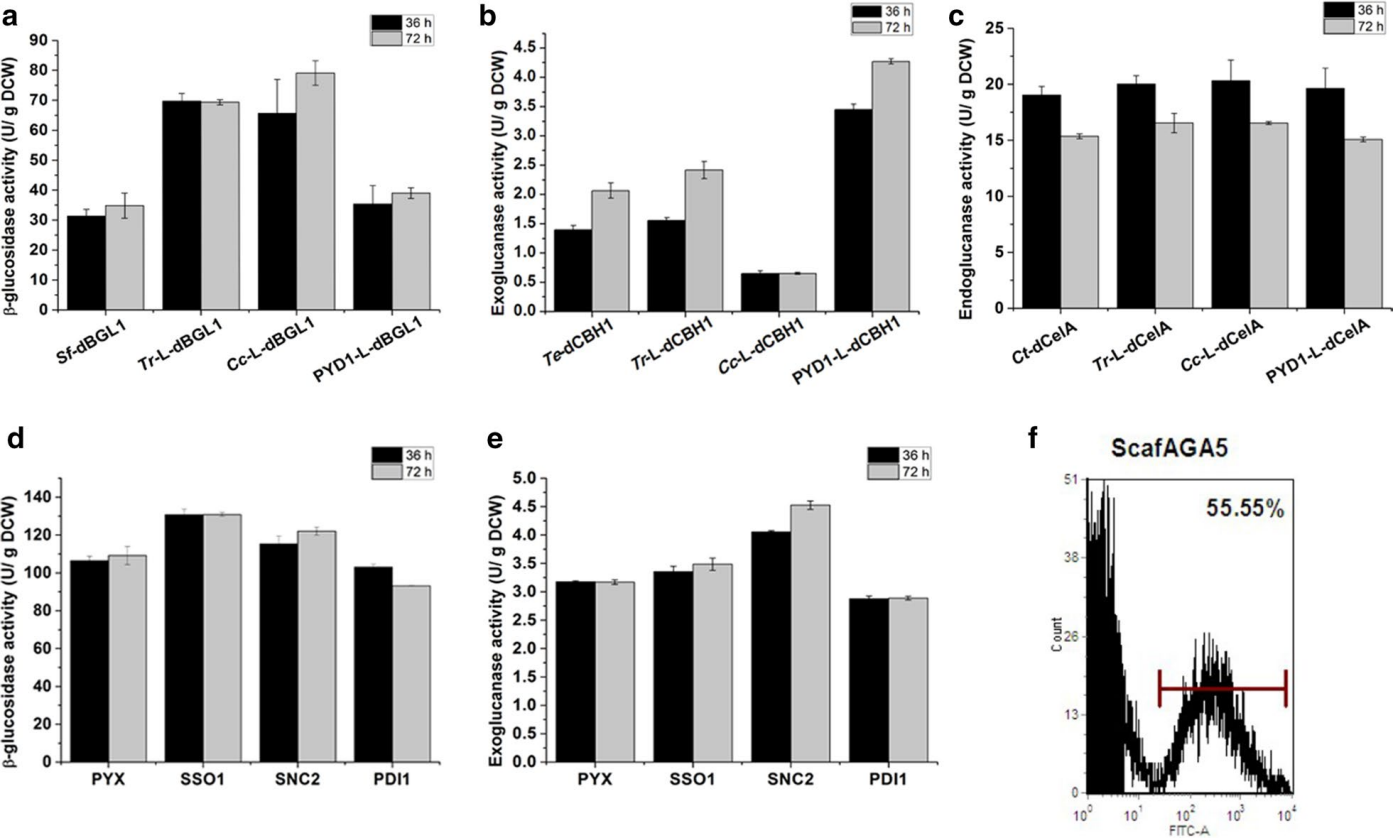
Improving the cellulase activity through linker optimization, engineering the secretory pathway and increasing the scaffoldin length for efficient cellulosome assembly. Activity of **a** β-glucosidase, **b** exoglucanase and **c** endoglucanase without and with different linkers between enzyme and dockerin. **d** Engineering secretory pathway improved *Tr*-L-dBGL1 extracellular activity. **e** Engineering secretory pathway improved PYD1-L-dBGL1 extracellular activity. **f** FACS analysis of surface display of scaffoldin ScafAGA5. Data were the mean values of two repeats ± standard deviation

Fusion with the dockerin domain decreased the extracellular activity of *Sf*-BGL1 significantly (Fig. 3a), perhaps because the dockerin domain interfered with the folding of *Sf*-BGL1 or the accessibility of the catalytic site. Therefore, three flexible linkers rich in serine or threonine (Additional file 4: Fig. S4) were added between dockerin and catalytic domains, respectively, to reduce possible interference. Naturally derived linkers from *T. reesei* CBH1 and *C. cellulovorans* EngB improved the extracellular activity of *Sf*-dBGL1 2.2- and 2.4-fold, respectively, while the synthetic linker from commercial plasmid pYD1 (Invitrogen) consisting of flexible unit GGGGS did not (Fig. 6a). For *Te*-dCBH1, the synthetic linker clearly increased the extracellular activity (2.1-fold), but the naturally derived linkers did not (Fig. 6b). None of the three linkers improved the extracellular activity of *Ct*-CelA (Fig. 6c). These results showed that different cellulosomal cellulases required different linkers for efficient secretion.

We recently showed that engineering the yeast secretory pathway including protein translocation, protein folding and vesicle trafficking can improve the extracellular activity of cellulases [28, 29]. Therefore, three key components in the secretory pathway including disulfide isomerase Pdi1p, t-SNARE (soluble *N*-ethylmaleimide-sensitive factor attachment receptor proteins) Sso1p and v-SNARE Snc2p were over-expressed to further improve the cellulosomal cellulase activity. Compared with control strain PYX which contained both enzyme expression and empty plasmid pYX242WS, Sso1p overexpression further increased the extracellular activity of *Tr*-L-dBGL1 by 19.8%, while Snc2p overexpression improved the extracellular activity of PYD1-L-dCBH1 by 45.1% (Fig. 6d, e). Besides, we have previously shown that Pdi1p overexpression enhanced the extracellular activity of *Ct*-CelA by 17% [29].

As the number of dockerins in the scaffoldin also affects the assembly efficiency, we created ScafAGA5, which has five tAag1p repeat units. The percentage of stained cells for the ScafAGA5 expressing strain was 55.6%, which was similar to the value for the ScafAGA3 expressing strain (Fig. 6f).

**Fig. 7:**
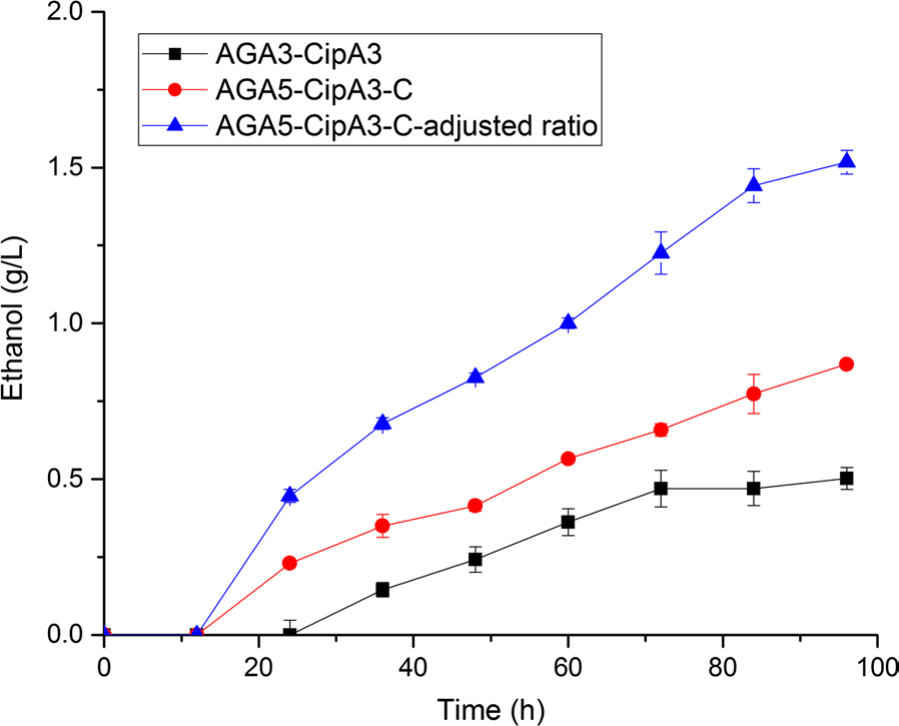
Direct ethanol production from PASC. Data are the mean values of two repeats ± standard deviation. AGA3–CipA3, cells containing both anchoring scaffoldin ScafAGA3 and scaffoldin I ScafCipA3 assembled complex cellulosome; AGA5–CipA3–C, cellulase activities were improved through linker optimization and secretory pathway engineering, and the cellulases were then assembled to the cells containing both anchoring scaffoldin ScafAGA5 and scaffoldin I ScafCipA3 to construct complex cellulosome; AGA5–CipA3–C-adjusted ratio, based on AGA5–CipA3–C, the ratio of ScafAGA5–ScafCipA3, CBH1, CelA and BGL1 was adjusted from 1:1:1:1 to 1:1.25:1.25:0.5

We then compared the ethanol production of PASC using the strains before and after optimization of the cellulase activity and scaffoldin length (Fig. 7). Without cellulase activity and scaffoldin length optimization, the strains displaying a complex cellulosome and containing two scaffoldins, ScafAGA3 and ScafCipA3, only produced 0.50 g/L ethanol, which was lower than for cells with a cellulosome containing only scaffoldin ScafAGA3 (0.89 g/L). Although more scaffoldin should increase the cellulase loading, the relatively low assembly capability of cellulases with ScafCipA3 in the complex cellulosome might counteract the beneficial impact of the increased scaffoldin number. Therefore, this complex cellulosome did not improve ethanol production. Cellulase secretion was optimized through linker optimization and secretion pathway engineering. Cells displaying a complex cellulosome with ScafAGA5 and ScafCipA3, and optimized by linker and secretion pathway, produced 0.87 g/L ethanol, which was 73% higher than cells without cellulase and scaffoldin optimization. Increasing the loading of CBH1 and CelA, and decreasing the loading of BGL1, is beneficial for ethanol production [30]. We therefore adjusted the ratio of ScafAGA5–ScafCipA3, CBH1, CelA and BGL1 containing cells from 1:1:1:1 to 1:1.25:1.25:0.5, which afforded an ethanol production of 1.52 g/L. Our results demonstrated that improving the cellulosome assembly efficiency and cellulase secretion is critical for increasing the fermentation capability. The ratio of different cellulases also needs to be optimized.

## Discussion

Several studies have shown that displaying cellulosomes on the *S. cerevisiae* cell surface can enable yeast to produce ethanol from cellulose [12–14, 31]. However, the application of yeast cellulosomes was limited by the low display efficiency of scaffoldins, the length of scaffoldins and the inefficient secretion of cellulases. To improve the performance of yeast cellulosome, the cell surface display was optimized by comparing various yeast cell wall proteins, scaffoldin was complicated by increasing cohesion number and utilizing double-layered scaffoldins, and better catalytic activity was obtained by improving enzyme activity and adjusting the cellulase ratio [12, 14–16]. After this engineering, most of the stains only produced about 1 or 2 g/L ethanol from PASC. Although higher ethanol production (3.5 g/L) was obtained, the cellulases were produced in *Escherichia coli* and then assembled on the yeast surface in vitro (Tsai et al. [13]). All these synthetic cellulosomes were assembled through hydrophobic interactions and hydrogen bonds, which have relatively low stability. In contrast, covalent bonds were more stable than these non-covalent bands. To investigate whether covalent bonds could be applied to construct yeast synthetic cellulosomes, using covalent disulfide bonds, which are involved in maintaining correct protein structure and forming multimeric proteins, was tested in this work [32, 33]. Fortunately, yeast cell wall protein a-agglutinin is a double-subunit protein [21]. Based on the a-agglutinin structure, we attempted to design Aga1p and Aga2p as a new cohesin and dockerin pair to assemble the cellulosome through disulfide bonds. Compared with traditional cellulosomes, the synthetic scaffoldins with repeating tAga1 units had a better display efficiency, while the synthetic cellulosomes had higher assembly efficiencies. Although the ethanol production was not as high as that of the previously reported cellulosome in yeast, the covalent bonds mediated cellulosome assembly improved the docking efficiency of the cellulases.

Based on previous studies, synthetic cellulosomes are a combination of multiple independent elements, including cohesin, dockerin and cellulases. Firstly, synthetic cellulosomes were constructed using cellulosomal cellulases which natively contained dockerin. Furthermore, free cellulases, which did not contain native dockerins, were fused to dockerins to successfully assemble cellulosome. Furthermore, immune proteins that interact with each other through non-covalent bonds have also been used as new cohesin–dockerin pair [14, 19]. Accordingly, scaffoldins have been further optimized by increasing the number of cohesion domains or using double-layered scaffoldins [12]. Therefore, we also integrated the new cohesin–dockerin pair into traditional cellulosomes. As shown in the results, scaffoldin consisting of five tAga1 s was displayed on the cell surface and used as the primary scaffoldin, and the traditional scaffoldin composed of three cohesins from *C. thermocellum* CipA was used as the secondary scaffoldin. The double-layered cellulosome was successfully assembled and applied to ethanol production.

We found that dockerin fusion significantly decreased the extracellular activity of *Sf*-BGL1, perhaps because the fusion of dockerin affects the structure of the enzyme. In a previous study, a linker between the two protein domains was required for the proteins to function well [34, 35]. Our results showed that a proper linker was required between the dockerin and catalytic domains, which could enhance the catalytic activity significantly. The secretion or display of heterologous proteins is often limited by protein misfolding and inefficient transport [36, 37]. Engineering the secretory pathway (including protein translocation, protein folding, glycosylation and vesicle trafficking) is an effective strategy for improving the production of heterologous proteins in *S. cerevisiae* [38]. Herein, we strengthened the protein folding and vesicle trafficking, resulting in a significant increase in the secretion of these cellulases. However, the cellulase activities were still relatively low. Therefore, engineering one or two components in the secretory pathway seems to only increase cellulase secretion slightly, with multiple engineering strategies remaining necessary to further increase protein secretion.

The co-incorporation of multiple enzymes in close proximity allows the direct transfer of intermediates among catalytic sites, reducing the diffusion of intermediates and significantly improving the catalytic efficiency [39]. Three dockerin-fused enzymes including AlsS, AlsD, and Bdh1 were assembled onto the scaffoldin, which was displayed on the yeast surface, and this multi-enzyme complex increased the 2,3-butanediol production clearly compared with free enzymes [40]. A cascade of redox enzymes involved in multiple electron-release through fuel oxidation was fused with a dockerin and then attached to the scaffoldin, with the multi-enzyme complex showing enhanced performance compared with traditional biofuel cells [41]. The tAga1–Aga2p pair in our work affords a new approach to the assembly of multi-enzyme complexes. Furthermore, other forms of covalent bonds could be explored to assemble the multi-enzyme complex. For instance, the protein that incorporates an unnatural amino acid (tyrosine analogues F_fact_) can react with another active cysteine-containing protein based on the covalent reaction between F_fact_ and cysteine [42]. The utilization of functional groups of unnatural amino acids may be another potential approach.

## Conclusions

In this study, a complex multiple-component assembly system constructed through disulfide bonds was developed for the first time. When applied to synthetic cellulosome construction, this system showed great advantages, such as increasing the display and assembly efficiency of the cellulosomes. This system provides multiple docking units for proteins and is suitable for expressing several synergistic enzymes, and will, therefore, have wide applications in synergistic catalysis.

## Methods

### Strains and media

*Escherichia coli* strain Trans2-blue (TransGene Biotech, Beijing, China) was used for plasmid propagation. *S. cerevisiae* CEN.PK102-5B (MATa; *URA*3-52, *HIS3*Δ1, *LEU2*-3,112) was used as the host for cellulosome construction [43]. *E. coli* was cultivated in Luria–Bertani (LB) medium (10 g/L tryptone, 5 g/L yeast extract, 10 g/L NaCl) with 100 μg/mL ampicillin. CEN.PK102-5B strain was grown in YPD medium (20 g/L tryptone, 5 g/L yeast extract, 20 g/L glucose). All recombinant yeast strains (Additional file 6: Table S2) were cultivated in SC–SCAA medium as reported previously without uracil, leucine or histidine [29, 44].

### Plasmid and strain construction

The primers and plasmids used in this work are shown in Additional file 5: TableS1 and Additional file 6: Table S2. Primers and genes were synthesized by Genewiz (Beijing, China). For cellulosome construction, expression vectors for scaffoldins and cellulases were constructed using the Gibson assembly [45]. Firstly, *AGA1* was amplified from the *S. cerevisiae* genome and cloned into plasmid pJFE3 [46] to construct pJFE3-AGA1. For the construction of a ScafCipA3 expression plasmid, the CipA3 fragment containing the first three cohesins and the CBD of scaffoldin CipA was amplified from *C. thermocellum* genomic DNA, and inserted into pYD1 to construct pYD1-CipA3. A fragment containing *AGA2*, *CipA3* and *V5 tag* was obtained by PCR from pYD1-CipA3 and ligated into pIYC04 [47] to form plasmid pIYC04-CipA3–AGA2. Finally, the fragment containing promoter *PGK1*, *AGA2*, *CipA3*, *V5 tag* and terminator *CYC1* was amplified and cloned into pJFE3-AGA1 to generate plasmid ScafCipA3. For construction of novel scaffoldin ScafAGA3, two tAga1p with a CBD from *Trichoderma reesei* CBH1 were synthesized and inserted into pJFE3 to afford pJFE3-2AGA1. *AGA1* without a signal peptide was amplified and inserted into pJFE3-2AGA1 to construct plasmid ScafAGA3. The C-terminal 687 bp of *SED1* was amplified and ligated into pJFE3-2AGA1 to afford plasmid 2AGA-SED. The genes encoding exoglucanases from *Talaromyces emersonii*, *H. grisea* and *C. thermophilum* (synthesized by Genewiz), endoglucanases from *Trichoderma reesei*, *C. thermocellum* and *C. cellulolyticum* (amplified from the corresponding genomic DNA) and β-glucosidase (amplified from plasmid pTH-BGL [29]) were fused with the corresponding dockerin derived from *C. thermocellum* CelS, CelA, and XynC and ligated into pIYC04. *C. thermocellum* endoglucanase CelA, *T. emersonii* exoglucanase CBHI, and *S. fibuligera* β-glucosidase BGL1 were fused with AGA2 and were also inserted in pIYC04. Linkers derived from *T. reesei* CBH1, *C. cellulovorans* EngB and commercial plasmid pYD1 were inserted between the dockerin domain and catalytic domain of the respective cellulosomal cellulases. The recombinant plasmids are described in Additional file 6: Table S2. These plasmids were transformed into *S. cerevisiae* CEN.PK102-5B and the resulting recombinant strains are shown in Additional file 7: Table S3.

### Enzymes activity measurement

The activity of cellobiohydrolase was quantified using *p*-nitrophenyl-β-d-cellobioside (*p*NPC) (Sigma, USA) as the substrate as described previously [48]. Enzymes were incubated in 50 mM citrate buffer (pH 4.8) with 2 mM *p*NPC at 50 °C for 30 min. The reaction was stopped by the addition of 10% sodium carbonate and *p*-nitrophenol (*p*NP) released from *p*NPC was determined at 405 nm. Endoglucanase activity was measured using carboxymethylcellulose sodium salt (CMC-Na; Sigma, USA) as the substrate [49]. Reducing sugars from hydrolyzed CMC-Na were boiled with dinitrosalicylate (DNS) for 10 min and then detected at 540 nm. β-Glucosidase activity was determined as described previously using *p*-nitrophenyl-β-d-glucopyranoside (*p*NPG; Sigma, USA) as the substrate [50]. Enzymes were mixed in 50 mM citrate buffer (pH 5.0) with 5 mM *p*NPG at 50 °C for 30 min. 10% sodium carbonate was added to stop the reaction and *p*NP released from *p*NPG was detected at 405 nm. One unit of the enzyme activity was defined as the amount of enzyme that released 1 μmol of product (*p*-nitrophenol or glucose) from the substrate at 50 °C in 1 min.

### Immunofluorescence assay and FACS analysis

*Saccharomyces cerevisiae* CEN.PK 102-5B with different phenotypes (Additional file 7: Table S3) were cultured for 24 h for analysis. To detect the assembly efficiency of traditional dockerin and cohesin, secreted cellulases with dockerin in the supernatant were concentrated about tenfold and incubated with strains expressing scaffoldin ScafCipA3 at an OD_600_ of 2, 5 and 15. The secreted cellulases incubated with ScafCipA3 expressing strains without concentration were used as controls. Cells were harvested and washed twice with phosphate-buffered saline solution (PBS, pH 7.0). The cells were then suspended in PBS containing 1 mg/mL bovine serum albumin to an OD_600_ of 1.0. Monoclonal mouse antibodies including anti-V5-FITC antibody (Invitrogen) for scaffoldin, anti-DDDDK tag antibody (DyLight 488; Abcam, UK) for BGL1 and CBHI, and anti-Myc tag antibody (fluorescein isothiocyanate conjugated [FITC]; Abcam, UK) for CelA were used for immunofluorescence microscopy and FACS analyses. The antibodies were mixed with the cell suspension at 25 °C for 1 h with 1:500 dilution. Cells were harvested and washed twice with PBS after staining. Images were taken using immunofluorescence microscopy (Olympus, Japan) and flow cytometry analysis (FACS) was performed using a FACSCanto II system (BD FACSCanto II, USA).

### Fermentation

Recombinant strains expressed exoglucanase, endoglucanase, β-glucosidase and different scaffoldins including ScafCipA3, ScafAGA3, both ScafAGA3 and ScafCipA3, and both ScafAGA5 and ScafCipA3, respectively. These strains were first pre-cultured in SC-SCAA medium at 30 °C for 48 h. The scaffoldin-displaying strains were incubated with the supernatant of cellulase-expressing strains at OD_600_ ratios of 1:1:1:1 or 1:1.25:1.25:0.5 for 4 h to allow cellulosome assembly. For ScafCipA3, the reaction was supplemented with 10 mM CaCl_2_. Cells with cellulosomes were washed twice and cultivated in YP medium with 1% phosphoric-acid-swollen cellulose (PASC) to an OD_600_ of 50. PASC was prepared from Avicel microcrystalline cellulose (Sheng Gong, China) as described previously [51]. Fermentation was performed anaerobically in 100-mL flasks containing 40 mL of culture. Samples (1 mL) were taken out periodically and the ethanol concentration was analyzed by HPLC using an Aminex HPX-87 H column (Bio-Rad, Richmond, CA) with 5 mmol/L H_2_SO_4_ as the mobile phase at a flow rate of 0.6 mL/min, at 45 °C. Peaks were detected using a RID-10A refractive index detector (Shimadzu, Kyoto, Japan).

## Additional files

**Additional file 1: Fig S1.** Functional test of tAGA1. A. The control strain expressed empty plasmids. B. The display levels of tAga1p fused with anchor protein Sed1p.

**Additional file 2: Fig S2.** FACS analysis of self-assembly of *Te*-dCBH1 (A), *Ct*-dCelA (B) and *Sf*-dBGL1 (C) on traditional scaffoldin ScafCipA3.

**Additional file 3: Fig S3.** Ethanol production directly from PASC.

**Additional file 4: Fig S4.** Sequences of three linkers.

**Additional file 5: Table S1.** Primers used in this study.

**Additional file 6: Table S2.** Plasmids used in this study.

**Additional file 7: Table S3.** Strains used in this study.
