# Supplementary material for: Efficient yeast surface-display of novel complex synthetic cellulosomes
Source: Microb Cell Fact. 2018 Aug 7;17:122. doi: 10.1186/s12934-018-0971-2 (PMC6081942; doi:10.1186/s12934-018-0971-2)
Supplement: Supplementary file 1 — Additional file 1: Fig S1. Functional test of tAGA1. A. The control strain expressed empty plasmids. B. The display levels of tAga1p fused with anchor protein Sed1p. [file 12934_2018_971_MOESM1_ESM.docx]

**Efficient yeast surface-display of novel complex synthetic cellulosomes**

Hongting Tang^1^, Jiajing Wang^1^, Shenghuan Wang^1^, Yu Shen^1^, Dina Petranovic^3^, Jin Hou^1^*, Xiaoming Bao^1,2^*

^1^State Key Laboratory of Microbial Technology, The College of Life Science, Shandong University, Jinan, 250100, China

^2^Shandong Provincial Key Laboratory of Microbial Engineering, Qi Lu University of Technology, Jinan 250353, PR China

^3^Department of Biology and Biological Engineering, Chalmers University of Technology, Kemivagen 10, Gothenburg SE-41296, Sweden.

* Corresponding author: Dr. Jin Hou, email: [houjin@sdu.edu.cn](mailto:houjin@sdu.edu.cn), Prof. Xiaoming Bao, email: [bxm@sdu.edu.cn](mailto:bxm@sdu.edu.cn); State Key Laboratory of Microbial Technology, The School of Life Science, Shandong University, Jinan 250100, China. Tel/ Fax: +86 531 8836 5826

Figure legends


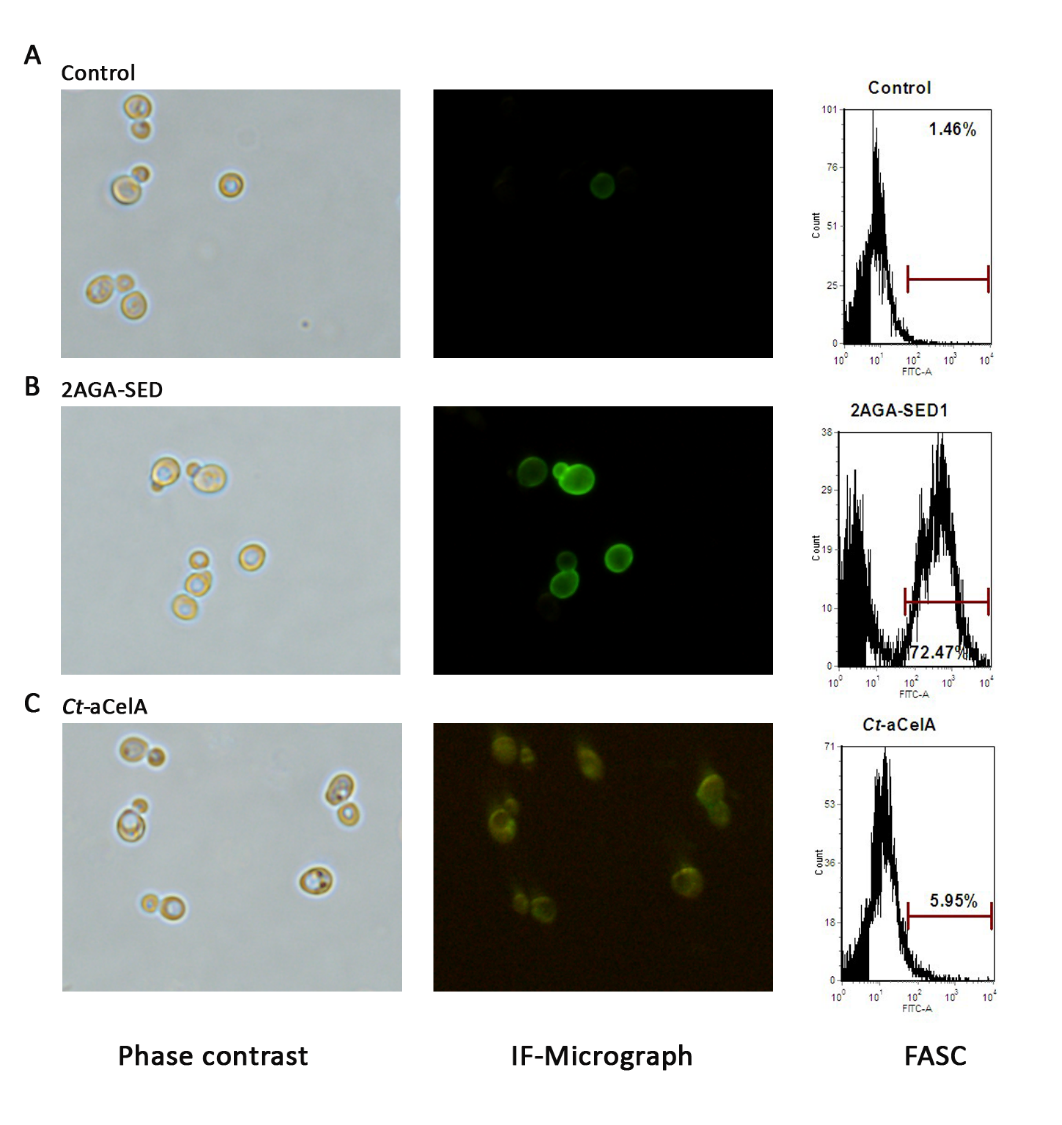


Fig. S1 Functional test of tAGA1. A. The control strain expressed empty plasmids. B. The display levels of tAga1p fused with anchor protein Sed1p. C. The self-assembly of *Ct*-aCelA on tAga1p-Sed1p. Control represented the strain without staining. The results are representative of two independent repeats.
