# Supplementary material for: Efficient yeast surface-display of novel complex synthetic cellulosomes
Source: Microb Cell Fact. 2018 Aug 7;17:122. doi: 10.1186/s12934-018-0971-2 (PMC6081942; doi:10.1186/s12934-018-0971-2)
Supplement: Supplementary file 4 — Additional file 4: Fig S4. Sequences of three linkers. [file 12934_2018_971_MOESM4_ESM.docx]

**Efficient yeast surface-display of novel complex synthetic cellulosomes**

Hongting Tang^1^, Jiajing Wang^1^, Shenghuan Wang^1^, Yu Shen^1^, Dina Petranovic^3^, Jin Hou^1^*, Xiaoming Bao^1,2^*

^1^State Key Laboratory of Microbial Technology, The College of Life Science, Shandong University, Jinan, 250100, China

^2^Shandong Provincial Key Laboratory of Microbial Engineering, Qi Lu University of Technology, Jinan 250353, PR China

^3^Department of Biology and Biological Engineering, Chalmers University of Technology, Kemivagen 10, Gothenburg SE-41296, Sweden.

* Corresponding author: Dr. Jin Hou, email: [houjin@sdu.edu.cn](mailto:houjin@sdu.edu.cn), Prof. Xiaoming Bao, email: [bxm@sdu.edu.cn](mailto:bxm@sdu.edu.cn); State Key Laboratory of Microbial Technology, The School of Life Science, Shandong University, Jinan 250100, China. Tel/ Fax: +86 531 8836 5826


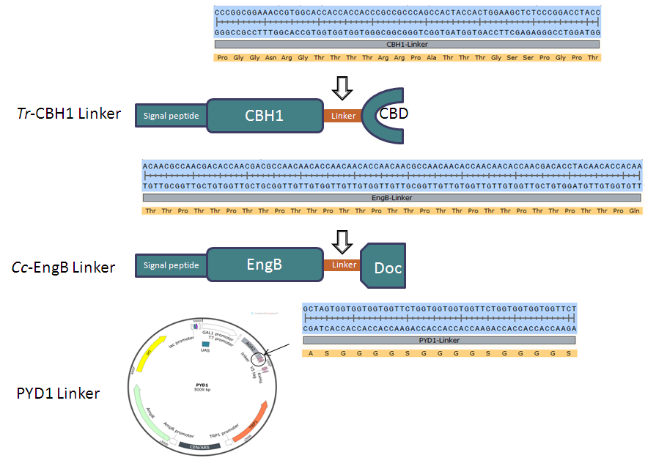


Fig. S4 Sequences of three linkers. *Tr*-CBH1 linker was derived from *T. reesei* CBH1. Cc-EngB linker was derived from *C. cellulovorans* EngB. PYD1 linker was from commercial plasmid PYD1.
