# Supplementary material for: Efficient yeast surface-display of novel complex synthetic cellulosomes
Source: Microb Cell Fact. 2018 Aug 7;17:122. doi: 10.1186/s12934-018-0971-2 (PMC6081942; doi:10.1186/s12934-018-0971-2)
Supplement: Supplementary file 5 — Additional file 5: Table S1. Primers used in this study. [file 12934_2018_971_MOESM5_ESM.docx]

**Efficient yeast surface-display of novel complex synthetic cellulosomes**

Hongting Tang^1^, Jiajing Wang^1^, Shenghuan Wang^1^, Yu Shen^1^, Dina Petranovic^3^, Jin Hou^1^*, Xiaoming Bao^1,2^*

^1^State Key Laboratory of Microbial Technology, The College of Life Science, Shandong University, Jinan, 250100, China

^2^Shandong Provincial Key Laboratory of Microbial Engineering, Qi Lu University of Technology, Jinan 250353, PR China

^3^Department of Biology and Biological Engineering, Chalmers University of Technology, Kemivagen 10, Gothenburg SE-41296, Sweden.

* Corresponding author: Dr. Jin Hou, email: [houjin@sdu.edu.cn](mailto:houjin@sdu.edu.cn), Prof. Xiaoming Bao, email: [bxm@sdu.edu.cn](mailto:bxm@sdu.edu.cn); State Key Laboratory of Microbial Technology, The School of Life Science, Shandong University, Jinan 250100, China. Tel/ Fax: +86 531 8836 5826

Table S1 Primers used in this study

| Primers | Sequences (5’ →3’) |
| --- | --- |
| Bsig-F | TCTTATCGTCGTCATCCTTGTAATCCATCGATACTAGTTCAAAGTTCTCTCAGAACGAG |
| Bsig-R | GAATTGGGACCTTGTCATCGTCGTCCTTGTAGTCAGCAACAGCAAGGCCTAGTG |
| BGL-F | GGCCTTGCTGTTGCTGACTACAAGGACGACGATGACAAGGTCCCAATTCAAAACTATAC |
| BGL1-R | GGCGGAACGCTGTTAACTATAGCATAAAATGCAGGTTTAATAGTAAACAGGACAGATG |
| XynC-F | TGTTAATGATATCAAGACATCTGTCCTGTTTACTATTAAACCTGCATTTTATGCTATAG |
| XynC-R | TCTTATCGTCGTCATCCTTGTAATCCATCGATACTAGTTCAAAGTTCTCTCAGAACGAG |
| SUC-F | AATTATCTACTTTTTACAACAAATATAAAACAAGGATCCATGCTTTTGCAAGCTTTCCT |
| tcSUC-R | GCCAGATCCTCTTCTGAGATGAGTTTTTGTTCTGCAGATATTTTGGCTGCAAAACCAGC |
| tcCelA-F | ATATCTGCAGAACAAAAACTCATCTCAGAAGAGGATCTGGCAGGTGTGCCTTTTAACAC |
| tcDOC-R | GTCTTCTTCGGAAATCAACTTCTGTTCCATGTCGACCTAATAAGGTAGGTGGGGTATG |
| trSUC-R | TGCAGATCCTCTTCTGAGATGAGTTTTTGTTCTGCAGATATTTTGGCTGCAAAACCAGC |
| trEG1-F | ATATCTGCAGAACAAAAACTCATCTCAGAAGAGGATCTGCAGCAACCGGGTACCAGCAC |
| trEG1-R | CATTACCGTCGCCATTTACATCAAGGCATTGCGAGTAGTAGTCGTTG |
| trDOC-F | CGACTACTACTCGCAATGCCTTGATGTAAATGGCGACGGTAATG |
| ccSUC-R | TACAGATCCTCTTCTGAGATGAGTTTTTGTTCTGCAGATATTTTGGCTGCAAAACCAGC |
| ccCelA-F | ATATCTGCAGAACAAAAACTCATCTCAGAAGAGGATCTGTATGATGCTTCACTTATTCC |
| ccCelA-R | CATTACCGTCGCCATTTACATCTCCATATACAATTACTGGGTC |
| ccDOC-F | GACCCAGTAATTGTATATGGAGATGTAAATGGCGACGGTAATG |
| bAGA2-F | AATCTAATCTAAGTTTTAATTACAAGCGGCCGCACTAGTATGCAGTTACTTCGCTGTT |
| bAGA2-R | CTTCCAGTGGTAGTGGCTGGGCGGCGGGTGGTGGTGGTGCCACGGTTTCCGCCGGGCCATGGAAAAACATACTGTGTGTTTATGG |
| BGL1-F | ACCACCACCACCCGCCGCCCAGCCACTACCACTGGAAGCTCTCCCGGACCTACCGTCCCAATTCAAAACTATACC |
| BGL1-R | CTTATCGTCGTCATCCTTGTAATCCATCGATACTAGTTCACTTGTCATCGTCGTCCTTG |
| aAGA2-F | TAATTATCTACTTTTTACAACAAATATAAAACAAGGATCCATGCAGTTACTTCGCTGTT |
| aAGA2-R | CTTCCAGTGGTAGTGGCTGGGCGGCGGGTGGTGGTGGTGCCACGGTTTCCGCCGGGGTCGACAAAAACATACTGTGTGTTTATGG |
| CelA-F | CCACCACCACCCGCCGCCCAGCCACTACCACTGGAAGCTCTCCCGGACCTACCGCAGGTGTGCCTTTTAACAC |
| CelA-R | GGATCTTAGCTAGCCGCGGTACCAAGCTTACTCGAGTCACAGATCCTCTTCTGAGATGAGTTTTTGTTCACCGTAAACAACCTGAGGAG |
| cAGA2-F | CTAATCTAAGTTTTAATTACAAGCGGCCGCACTAGTATGCAGTTACTTCGCTGTTTTTC |
| cAGA2-R | CTGGAAGCTCTCCCGGACCTACCCAGCAAGCTGGTACAGCTAC |
| CBH1-F | CTGGAAGCTCTCCCGGACCTACCCAGCAAGCTGGTACAGCTAC |
| CBH1-R | TATCGTCGTCATCCTTGTAATCCATCGATACTAGTTTATGAAGCTGTAAATGTAGAGTT |
| AGA1-F | AATCTAATCTAAGTTTTAATTACAAAGGATCCTCTAGAATGACATTATCTTTCGCTCAT |
| AGA1-R | TTTCAATTCAATTCAATCCTGCAGGTCGACTCTAGATTAACTGAAAATTACATTGCAAG |
| CIPA3-F | AATTCTGCAGATATCCAGCACAGTGGCGGCCGCTCGAGGCCACAATGACAGTCGAGATC |
| CIPA3-R | GGGATAGGCTTACCTTCGAAGGGCCCTCTAGACTCGAGATTCGAATCATCTGTCGGTG |
| AGA2-CIPA3-F | AATTATCTACTTTTTACAACAAATATAAAACAAGGATCCATGCAGTTACTTCGCTGTT |
| AGA2-CIPA3-R | CGGATCTTAGCTAGCCGCGGTACCAAGCTTACTCGAGTCAATGGTGATGGTGATGATG |
| PGK-F | CCCAGTCACGACGTTGTAAAACGACGGCCAGTGAATTCGGAAGTACCTTCAAAGAATGG |
| CYC-R | CGTACAAAGTATGCATTGTGGTACCGAGCTCGAATTCCTTCGAGCGTCCCAAAACCTTC |
| SED1-F | ACTGCTAGCCTCGAGGGATCCTCTAGAGTCGACCTGCAGGCTCTTCCAACTAACGGTAC |
| SED1-R | TGATCTATCGATTTCAATTCAATTCAATCCTGCAGTCATAAGAATAACATAGCAACACC |
| Tr-L-BGL1-R | CCACCACCCGCCGCCCAGCCACTACCACTGGAAGCTCTCCCGGACCTACCAAACCTGCATTTTATGCTATAG |
| Tr-L-Bdoc-F | CGGGAGAGCTTCCAGTGGTAGTGGCTGGGCGGCGGGTGGTGGTGGTGCCACGGTTTCCGCCGGGAATAGTAAACAGGACAGATGTC |
| PYD-L-BGL1-R | CAGAACCACCACCACCAGAACCACCACCACCACTAGCAATAGTAAACAGGACAGATGTC |
| PYD-L-Bdoc-F | TGGTTCTGGTGGTGGTGGTTCTGGTGGTGGTGGTTCTAAACCTGCATTTTATGCTATAG |
| Cc-L-BGL1-R | TGTTGGTGTTGTTGGCGTTGTTGGTGTTGTTGGTGTTGTTGGCGTCGTTGGTGTCGTTGGCGTTGTAATAGTAAACAGGACAGATGTC |
| Cc-L-Bdoc-F | CAACACCAACAACACCAACAACGCCAACAACACCAACAACACCAACGACACCTACAACACCACAAAAACCTGCATTTTATGCTATAG |
| Tr-L-CelA-R | CCGGGAGAGCTTCCAGTGGTAGTGGCTGGGCGGCGGGTGGTGGTGGTGCCACGGTTTCCGCCGGGACCGTAAACAACCTGAGGAG |
| Tr-L-Adoc-F | CCAGCCACTACCACTGGAAGCTCTCCCGGACCTACCGATGTAAATGGCGACGGTAATG |
| PYD-L-CelA-R | CCAGAACCACCACCACCAGAACCACCACCACCACTAGCACCGTAAACAACCTGAGGAG |
| PYD-L-Adoc-F | GGTTCTGGTGGTGGTGGTTCTGGTGGTGGTGGTTCTGATGTAAATGGCGACGGTAATG |
| Cc-L-CelA-R | GTTGGTGTTGTTGGCGTTGTTGGTGTTGTTGGTGTTGTTGGCGTCGTTGGTGTCGTTGGCGTTGTACCGTAAACAACCTGAGGAG |
| Cc-L-Adoc-F | CAACACCAACAACACCAACAACGCCAACAACACCAACAACACCAACGACACCTACAACACCACAAGATGTAAATGGCGACGGTAATG |
| Tr-L-CBH1-R | GGTGGTTCTGGTGGTGGTGGTTCTGGTGGTGGTGGTTCTCCATGGGGTTCTGGTGGTGG |
| Tr-L-Cdoc-F | CACCAGAACCACCACCACCAGAACCACCACCACCACTAGCTGAAGCTGTAAATGTAGAG |
| PYD-L-CBH1-R | GCCCAGCCACTACCACTGGAAGCTCTCCCGGACCTACCCCATGGGGTTCTGGTGGTGG |
| PYD-L-Cdoc-F | GAGCTTCCAGTGGTAGTGGCTGGGCGGCGGGTGGTGGTGGTGCCACGGTTTCCGCCGGGTGAAGCTGTAAATGTAGAG |
| Cc-L-CBH1-R | GTGTTGTTGGTGTTGTTGGCGTTGTTGGTGTTGTTGGTGTTGTTGGCGTCGTTGGTGTCGTTGGCGTTGTTGAAGCTGTAAATGTAGAG |
| Cc-L-Cdoc-F | CAACAACACCAACAACACCAACAACGCCAACAACACCAACAACACCAACGACACCTACAACACCACAACCATGGGGTTCTGGTGGTGG |
