# Supplementary material for: Efficient yeast surface-display of novel complex synthetic cellulosomes
Source: Microb Cell Fact. 2018 Aug 7;17:122. doi: 10.1186/s12934-018-0971-2 (PMC6081942; doi:10.1186/s12934-018-0971-2)
Supplement: Supplementary file 6 — Additional file 6: Table S2. Plasmids used in this study. [file 12934_2018_971_MOESM6_ESM.docx]

**Efficient yeast surface-display of novel complex synthetic cellulosomes**

Hongting Tang^1^, Jiajing Wang^1^, Shenghuan Wang^1^, Yu Shen^1^, Dina Petranovic^3^, Jin Hou^1^*, Xiaoming Bao^1,2^*

^1^State Key Laboratory of Microbial Technology, The College of Life Science, Shandong University, Jinan, 250100, China

^2^Shandong Provincial Key Laboratory of Microbial Engineering, Qi Lu University of Technology, Jinan 250353, PR China

^3^Department of Biology and Biological Engineering, Chalmers University of Technology, Kemivagen 10, Gothenburg SE-41296, Sweden.

* Corresponding author: Dr. Jin Hou, email: [houjin@sdu.edu.cn](mailto:houjin@sdu.edu.cn), Prof. Xiaoming Bao, email: [bxm@sdu.edu.cn](mailto:bxm@sdu.edu.cn); State Key Laboratory of Microbial Technology, The School of Life Science, Shandong University, Jinan 250100, China. Tel/ Fax: +86 531 8836 5826

Table S2 Plasmids used in this study

| Plasmid | Genotype | Reference |
| --- | --- | --- |
| pIYC04 | Yeast 2μ plasmid with *HIS3* marker | 37 |
| pJEF3 | Yeast 2μ plasmid with *URA3* marker | 36 |
| Sf-dBGL1 | pIYC04 with *TEF1* promoter, *ADH1* terminator and *S. fibuligera* *BGL1* with the dockerin derived from *C. thermocellum* *XynC* | This study |
| Ct-dCelA | pIYC04 with *PGK1* promoter,*CYC1* terminator and *C. thermocellum* *CelA* | This study |
| Cc-dCelA | pIYC04 with *PGK1* promoter,*CYC1* terminator and *CelA* gene from *C. cellulolyticum* with the dockerin derived from *C. thermocellum* *CelA* | This study |
| Tr-dEG1 | pIYC04 with *PGK1* promoter,*CYC1* terminator and *CelA* gene from *T. reesei* with the dockerin derived from *C. thermocellum* *CelA* | This study |
| Te-dCBH1 | pIYC04 with *TEF1* promoter, *ADH1* terminator and *T. emersonii* *CBH1* with the dockerin of CelS from *C. thermocellum* | This study |
| Hg-dCBH1 | pIYC04 with *TEF1* promoter, *ADH1* terminator and *H. grisea* *CBH1* with the dockerin derived from *C. thermocellum* *CelS* | This study |
| Ct-dCBH1 | pIYC04 with *TEF1* promoter, *ADH1* terminator and *C. thermophilum* *CBH1* with the dockerin derived from *C. thermocellum* *CelS* | This study |
| Sf-aBGL1 | pIYC04 with *TEF1* promoter, *ADH1* terminator and *S. fibuligera BGL1* fused with *AGA2* | This study |
| Ct-aCelA | pIYC04 with *PGK1* promoter,*CYC1* terminator and *C. thermocellum CelA* fused with *AGA2* | This study |
| Te-aCBH1 | pIYC04 with *TEF1* promoter, *ADH1* terminator and *T. emersonii CBH1* fused with *AGA2* | This study |
| aBGL-aCelA | pIYC04 with *TEF1* promoter, *ADH1* terminator and *S. fibuligera BGL1* fused with *AGA2*, and with *PGK1* promoter,*CYC1* terminator and *C. thermocellum CelA* fused with *AGA2* | This study |
| pIYC04-CipA3-AGA2 | pIYC04 with *TEF1* promoter, *PGK1* terminator and *AGA1* gene and with *PGK1* promoter, *CYC1* terminator and *CipA*3 derived from *C. thermocellum* *CipA* fused with *AGA2* | This study |
| ScafCipA3 | pJFE3 with *TEF1* promoter, *PGK1* terminator and *AGA1* gene and with *PGK1* promoter, *CYC1* terminator and *CipA*3 derived from *C. thermocellum* *CipA* fused with *AGA2* | This study |
| 2AGA-SED | pJFE3 with *TEF1* promoter, *PGK1* terminator and t*AGA1* with the GPI domain of *SED1* | This study |
| ScafAGA3 | pJFE3 with *TEF1* promoter, *PGK1* terminator and scaffoldin t*AGA*3 with the GPI domain of *AGA1* | This study |
| ScafAGA5 | pJFE3 with *TEF1* promoter, *PGK1* terminator and scaffoldin t*AGA*5 with the GPI domain of *AGA1* | This study |
| Tr-l-dBGL1 | pIYC04 with *TEF1* promoter, *ADH1* terminator and *Sf-*d*BGL1* with linker derived from *T. reesei* *Cel7A* | This study |
| Cc-l-dBGL1 | pIYC04 with *TEF1* promoter, *ADH1* terminator and *Sf-*d*BGL1* with linker derived from *C. cellulovorans* *EngB* | This study |
| PYD-l-dBGL1 | pIYC04 with *TEF1* promoter, *ADH1* terminator and *Sf-*d*BGL1* with linker derived from PYD1 | This study |
| Tr-l-dCelA | pIYC04 with *PGK1* promoter,*CYC1* terminator and *Ct-*d*CelA* with linker derived from *T. reesei* *Cel7A* | This study |
| Cc-l-dCelA | pIYC04 with *PGK1* promoter,*CYC1* terminator and *Ct-*d*CelA* with linker derived from *C. cellulovorans* *EngB* | This study |
| PYD-l-dCelA | pIYC04 with *PGK1* promoter,*CYC1* terminator and *Ct-*d*CelA* with linker derived from PYD1 | This study |
| Tr-l-dCBH1 | pIYC04 with *TEF1* promoter, *ADH1* terminator and *Te-*d*CBH1* with linker derived from *T. reesei* *Cel7A* | This study |
| Cc-l-dCBH1 | pIYC04 with *TEF1* promoter, *ADH1* terminator and *Te-*d*CBH1* with linker derived from *C. cellulovorans* *EngB* | This study |
| PYD-l-dCBH1 | pIYC04 with *TEF1* promoter, *ADH1* terminator and *Te-*d*CBH1* with linker derived from PYD1 | This study |
| pTHX-PDI1 | PYX242WS with *TPI1* promoter, *PGK1* terminator and *PDI1* gene | (Tang *et al.*, 2015) |
| SSO1 | pYX242WS with *TEF1* promoter, *PolyA* terminator and *SSO1* gene | (Tang *et al.*, 2017) |
| SNC2 | pYX242WS with *TEF1* promoter, *PolyA* terminator and *SNC2* gene | (Tang *et al.*, 2017) |
