# Supplementary material for: Efficient yeast surface-display of novel complex synthetic cellulosomes
Source: Microb Cell Fact. 2018 Aug 7;17:122. doi: 10.1186/s12934-018-0971-2 (PMC6081942; doi:10.1186/s12934-018-0971-2)
Supplement: Supplementary file 7 — Additional file 7: Table S3. Strains used in this study. [file 12934_2018_971_MOESM7_ESM.docx]

**Efficient yeast surface-display of novel complex synthetic cellulosomes**

Hongting Tang^1^, Jiajing Wang^1^, Shenghuan Wang^1^, Yu Shen^1^, Dina Petranovic^3^, Jin Hou^1^*, Xiaoming Bao^1,2^*

^1^State Key Laboratory of Microbial Technology, The College of Life Science, Shandong University, Jinan, 250100, China

^2^Shandong Provincial Key Laboratory of Microbial Engineering, Qi Lu University of Technology, Jinan 250353, PR China

^3^Department of Biology and Biological Engineering, Chalmers University of Technology, Kemivagen 10, Gothenburg SE-41296, Sweden.

* Corresponding author: Dr. Jin Hou, email: [houjin@sdu.edu.cn](mailto:houjin@sdu.edu.cn), Prof. Xiaoming Bao, email: [bxm@sdu.edu.cn](mailto:bxm@sdu.edu.cn); State Key Laboratory of Microbial Technology, The School of Life Science, Shandong University, Jinan 250100, China. Tel/ Fax: +86 531 8836 5826

Table S3 Strains used in this study

| Strains | Plasmid(s) | Phenotype |
| --- | --- | --- |
| pJFE3 | pJFE3 | No protein expression |
| CipA3 | ScafCipA3 | Display scaffoldin ScafCipA3 on cell surface |
| AGA3 | ScafAGA3 | Display scaffoldin ScafAGA3 on cell surface |
| AGA5 | ScafAGA5 | Display scaffoldin ScafAGA5 on cell surface |
| A3C3 | ScafAGA3; pIYC04-CipA3-AGA2 | Display scaffoldin ScafAGA3 and ScafCipA3 on cell surface |
| A5C3 | ScafAGA5; pIYC04-CipA3-AGA2 | Display scaffoldin ScafAGA5 and ScafCipA3 on cell surface |
| *Sf*-dBGL1 | *Sf*-dBGL1 | Secretion of *Sf*-dBGL1 |
| *Ct*-dCelA | *Ct*-dCelA | Secretion of *Ct*-dCelA |
| *Cc*-dCelA | *Cc*-dCelA | Secretion of *Cc*-dCelA |
| *Tr*-dEGI | *Tr*-dEGI | Secretion of *Tr*-dEGI |
| *Te*-dCBH1 | *Te*-dCBH1 | Secretion of *Te*-dCBH1 |
| *Hg*-dCBH1 | *Hg*-dCBH1 | Secretion of *Hg*-dCBH1 |
| *Ct*-dCBH1 | *Ct*-dCBH1 | Secretion of *Ct*-dCBH1 |
| *Sf*-aBGL1 | *Sf*-aBGL1 | Secretion of *Sf*-aBGL1 |
| *Ct*-aCelA | *Ct*-aCelA | Secretion of *Ct*-aCelA |
| *Te*-aCBH1 | *Te*-aCBH1 | Secretion of *Te*-aCBH1 |
| CBGL1 | ScafCipA3, *Sf*-dBGL1 | Assembly *Sf*-dBGL1on surface-display ScafCipA3 |
| CCelA | ScafCipA3, *Ct*-dCelA | Assembly *Ct*-dCelA on surface-display ScafCipA3 |
| CCBH1 | ScafCipA3, *Te*-dCBH1 | Assembly *Te*-dCBH1 on surface-display ScafCipA3 |
| ABGL1 | ScafAGA3, *Sf*-aBGL1 | Assembly *Sf*-aBGL1 on surface-display ScafAGA3 |
| ACelA | ScafAGA3, *Ct*-aCelA | Assembly *Ct*-aCelA on surface-display ScafAGA3 |
| ACBH1 | ScafAGA3, *Te*-aCBH1 | Assembly *Te*-aCBH1 on surface-display ScafAGA3 |
| ABCC | ScafAGA3-aCBH1, aBGL-aCelA | Assembly *Sf*-aBGL1, *Ct*-aCelA and *Te*-aCBH1 on surface-display ScafAGA3 |
| Tr-L-dBGL1 | Tr-l-dBGL1 | Secretion of *Sf*-dBGL1 with linker derived from *T. reesei* CBH1 |
| Cc-L-dBGL1 | Cc-l-dBGL1 | Secretion of *Sf*-dBGL1 with linker derived from *C. cellulovorans* EngB |
| PYD-L-dBGL1 | PYD-l-dBGL1 | Secretion of *Sf*-dBGL1 with linker derived from PYD1 |
| Tr-L-dCelA | Tr-l-dCelA | Secretion of *Ct*-dCelA with linker derived from *T. reesei* CBH1 |
| Cc-L-dCelA | Cc-l-dCelA | Secretion of *Ct*-dCelA with linker derived from *C. cellulovorans* EngB |
| PYD-L-dCelA | PYD-l-dCelA | Secretion of *Ct*-dCelA with linker derived from PYD1 |
| Tr-L-dCBH1 | Tr-l-dCBH1 | Secretion of *Te*-dCBH1 with linker derived from *T. reesei* CBH1 |
| Cc-L-dCBH1 | Cc-l-dCBH1 | Secretion of *Te*-dCBH1 with linker derived from *C. cellulovorans* EngB |
| PYD-L-dCBH1 | PYD-l-dCBH1 | Secretion of *Te*-dCBH1 with linker derived from PYD1 |
| L-dBGL1-Sso1p | Tr-l-dBGL1; SSO1 | Secretion of *Sf*-dBGL1 with linker derived from *T. reesei* CBH1 in Sso1p expressing strain |
| L-dBGL1-Snc2p | Tr-l-dBGL1; SNC2 | Secretion of *Sf*-dBGL1 with linker derived from *T. reesei* CBH1 in Snc2p expressing strain |
| L-dBGL1-Pdi1p | Tr-l-dBGL1; pTHX-PDI1 | Secretion of *Sf*-dBGL1 with linker derived from *T. reesei* CBH1 in Pdi1p expressing strain |
| L-dCBH1-Sso1p | PYD-l-dCBH1; SSO1 | Secretion of *Te*-dCBH1 with linker derived from PYD1 in Sso1p expressing strain |
| L-dCBH1-Snc2p | PYD-l-dCBH1; SNC2 | Secretion of *Te*-dCBH1 with linker derived from PYD1 in Snc2p expressing strain |
| L-dCBH1-Pdi1p | PYD-l-dCBH1; pTHX-PDI1 | Secretion of *Te*-dCBH1 with linker derived from PYD1 in Pdi1p expressing strain |
